# Implementing the sterile insect technique with RNA interference – a review

**DOI:** 10.1111/eea.12575

**Published:** 2017-06-23

**Authors:** Michael Darrington, Tamas Dalmay, Neil I. Morrison, Tracey Chapman

**Affiliations:** ^1^ School of Biological Sciences University of East Anglia Norwich Research Park Norwich Norfolk NR4 7TJ UK; ^2^ Oxitec Limited 71 Innovation Drive, Milton Park Oxford OX14 4RQ UK

**Keywords:** insect control, environmental RNAi, SIT, non‐GM pest control, double‐stranded RNA

## Abstract

We review RNA interference (RNAi) of insect pests and its potential for implementing sterile insect technique (SIT)‐related control. The molecular mechanisms that support RNAi in pest species are reviewed in detail, drawing on literature from a range of species including *Drosophila melanogaster* Meigen and *Homo sapiens* L. The underlying genes that enable RNAi are generally conserved across taxa, although variance exists in both their form and function. RNAi represents a plausible, non‐GM system for targeting populations of insects for control purposes, if RNAi effector molecules can be delivered environmentally (eRNAi). We consider studies of eRNAi from across several insect orders and review to what extent taxonomy, genetics, and differing methods of double‐stranded (ds) RNA synthesis and delivery can influence the efficiency of gene knockdown. Several factors, including the secondary structure of the target mRNA and the specific nucleotide sequence of dsRNA effector molecules, can affect the potency of eRNAi. However, taxonomic relationships between insects cannot be used to reliably forecast the efficiency of an eRNAi response. The mechanisms by which insects acquire dsRNA from their environment require further research, but the evidence to date suggests that endocytosis and transport channels both play key roles. Delivery of RNA molecules packaged in intermediary carriers such as bacteria or nanoparticles may facilitate their entry into and through the gut, and enable the evasion of host defence systems, such as toxic pH, that would otherwise attenuate the potential for RNAi.

## RNAi and the sterile insect technique (SIT)

Established methods of insect control are under continual review and development in order to keep track of new knowledge, changing legislation, regulatory concerns, and the maintenance of efficacy (e.g., in the face of increased resistance to pesticides) (Gross, 2013; Tabashnik et al., 2014). In this context, the development of new methods for insect control is of key importance and there has been intense interest in the utility of gene silencing methods induced by RNA interference (RNAi). RNAi can induce mortality (Yang & Han, 2014; Cao et al., 2015; Abd El Halim et al., 2016; Christiaens et al., 2016; Hu et al., 2016; Malik et al., 2016), create beneficial phenotypes for insect control (Salvemini et al., 2009; Shukla & Palli, 2012; Peng et al., 2015; Yu et al., 2016), and prevent pesticide resistance in insect pests (Figueira‐Mansur et al., 2013; Guo et al., 2015; Wei et al., 2015; Bona et al., 2016; Sandoval‐Mojica & Scharf, 2016). Therefore, the potential for RNAi as a basis for future pest management strategies holds great promise (Huvenne & Smagghe, 2010; Gu & Knipple, 2013; Scott et al., 2013; Baum & Roberts, 2014; Kim et al., 2015). The purpose of this review is to summarize the mechanisms by which gene silencing is achieved, describe the ways in which it is currently being used, and to explore the many factors that affect the efficacy of RNAi in this context.

**Figure 1:**
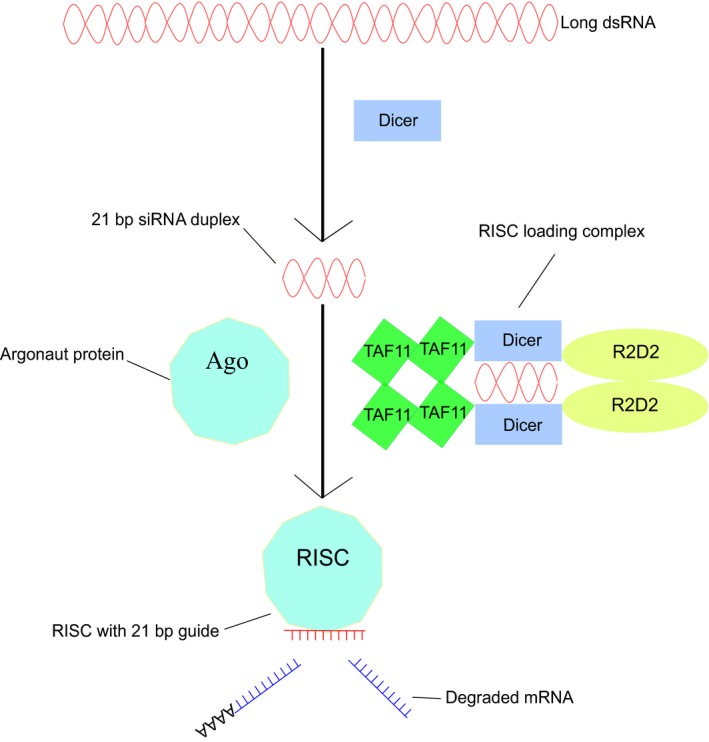
The canonical siRNA pathway. Cytoplasmic long double‐stranded RNAs are processed into 21‐bp duplex siRNAs by Dicer endonucleases. Dicer then complexes with various molecules to form a RISC loading complex (RLC) (the proposed RLC variant found in *Drosophila melanogaster* is shown here; Liang et al., 2015). The RLC introduces siRNA to an Argonaute protein, which degrades a single ‘passenger’ strand of the duplex, whilst binding its cognate partner to form an RNA induced silencing complex (RISC). The RISC then utilizes the nucleotide sequence of the bound ‘guide’ strand to scan cellular mRNAs, which it targets for knockdown via degradation.

RNAi can be used to achieve knock‐down of the level of gene expression in specific target genes. This is done via the introduction, by various means, of double‐stranded RNA (dsRNA) into the cells of the target species (Fire et al., 1998). The evidence suggests that RNAi is facilitated by the canonical small interfering RNA (siRNA) pathway, which results in mRNA degradation (Figure 1). In our review of the mechanisms of RNAi in pest insects we draw strongly from the well‐described canonical siRNA pathway in *Drosophila melanogaster* Meigen and *Homo sapiens* L.

It has been increasingly realized that a classic method of insect control, the sterile insect technique (SIT) (Knipling, 1955) could, in principle, be implemented through RNAi (Whyard et al., 2015). The SIT relies upon the production of large numbers of sterile insects for release (usually males) that subsequently mate with wild individuals, resulting in sterile matings and a reduction in the pest population size (Knipling, 1998; Krafsur, 1998). The key to SIT is the effective production of large numbers of sterile individuals. This crucial step is also a potential weakness of the approach. For example, the induction of sterility through irradiation results in well‐documented costs to insect performance, and hence control potential (Hooper, 1972; Toledo et al., 2004; Guerfali et al., 2011). Newer developments based on SIT that avoid irradiation, e.g., genetically engineered ‘self‐limiting’ insects (Thomas et al., 2000), can be highly effective (Harris et al., 2011; Carvalho et al., 2015; Gorman et al., 2016) but rely upon the release of genetically engineered insects, which may not be possible in all countries.

The principles by which RNAi might offer an alternative route for the induction of sterility, as well as other potentially useful manipulations for insect control, were recently investigated in a study using *Aedes aegypti* (L.) (Whyard et al., 2015). The scenario envisaged by Whyard et al. (2015) requires knockdown of at least two genes in the target insects. First, females would be targeted through silencing of a gene in the sexual differentiation cascade to turn them into pseudomales, i.e., genetic females which are phenotypically male (Pane et al., 2002; Salvemini et al., 2009; Shukla & Palli, 2012; Liu et al., 2015). Next, genes that could induce male (and pseudomale) sterility would be targeted in order to produce a 100% sterile male release cohort (Whyard et al., 2015). However, two equally important conditions must be met before this technique can be applied in the field, as described below.

The primary condition of RNAi‐based SIT is that the sex reversal target must reliably produce a male‐only cohort. There are clear benefits of releasing only one sex in SIT programmes, for example it can avoid both assortative mating between released insects and any pest‐related damage caused by females. The second condition is to ensure that silencing of neither the sex reversal nor the sterility target unduly reduces insect performance. Evidence suggests that these conditions can be met, although further supporting research is required.

Through RNAi of transformer‐2, Salvemini et al. (2009) were able to produce a *Ceratitis capitata* (Wiedemann) cohort which was 95.6% phenotypically male. Karyotypic analysis of phenotypically male flies (n = 20) demonstrated that they were 55% genetically female. Most importantly, pseudomales were observed completing male‐specific courtship rituals, which should allow them to attract and copulate with females (Briceño & Eberhard, 2003). Gabrieli et al. (2016) report that RNAi of innexin‐5 in *C. capitata* produced spermless, sterile males. Spermless males remained sexually competitive with wild‐type rivals and were able to induce similar post‐mating responses. It is possible that simultaneous RNAi of transformer‐2 and innexin‐5 (or conserved homologous genes in diverse species) could produce a male‐only, sterile cohort that could be used for SIT. However, it is important to note that simultaneous gene silencing is unpredictable (Table 2) and that Gabrieli et al. (2016) and Salvemini et al. (2009) microinjected insect eggs with dsRNA, a technique that is incompatible with large‐scale SIT.

Microinjection of dsRNA has been demonstrated to induce RNAi in several insects (Paim et al., 2013; Peng et al., 2015; Xue et al., 2015; Yu et al., 2016). However, SIT programmes may require the production and release of up to a billion insects per week (Alphey et al., 2010) and injection techniques cannot be used to treat insects in such numbers. Therefore, RNAi may provide a useful tool for implementing SIT if gene silencing can be induced via environmental dsRNA (eRNAi) (Whangbo & Hunter, 2008).

**Figure 2:**
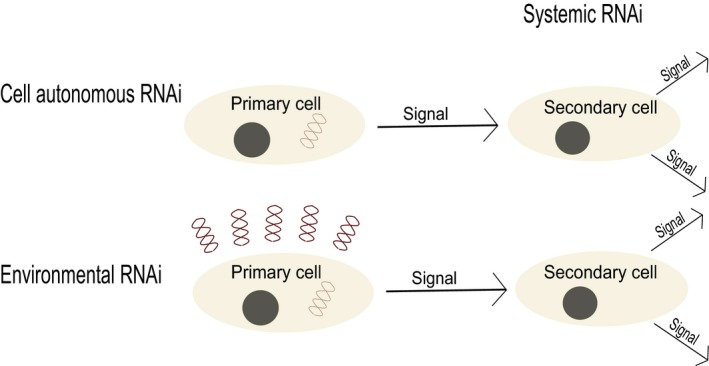
Categories of RNAi response. Cell autonomous RNAi is gene silencing in response to cytoplasmic dsRNA of viral or experimental origin. Non‐cell autonomous RNAi occurs in response to an extracellular signal, and is subcategorized by the origin of that signal as either environmental (eRNAi), or systematic RNAi. eRNAi occurs when a cell takes up environmental dsRNA molecules and elicits a gene silencing response. Systemic RNAi is initiated in a secondary cell when a silencing signal is received from a primary cell. Systemic RNAi can be a by‐product of either non‐cell autonomous RNAi or eRNAi in a primary cell, and if the secondary cell further propagates the signal, this can induce global gene silencing.

Cell autonomous RNAi defines gene silencing in response to intracellular dsRNA of experimental or viral origin. Non‐cell autonomous RNAi defines gene silencing in response to an extracellular signal, and is further divided into systemic RNAi or eRNAi based on the nature of that signal. eRNAi describes gene silencing in response to proximal dsRNA molecules, whereas systemic describes RNAi gene silencing in response to a signal received from a proximal cell. Therefore, both non‐cell autonomous RNAi and eRNAi occur in a primary cell in direct response to dsRNA, whereas systemic RNAi is initiated in a secondary cell in response to an as yet undefined signal received from a primary cell (Figure 2). eRNAi can be achieved via the introduction of dsRNAs via food (Asokan et al., 2014; Coleman et al., 2015; Li et al., 2015b; Sandoval‐Mojica & Scharf, 2016) or through topical delivery (Toprak et al., 2013; Whyard et al., 2015). For reasons that are as yet not entirely clear, the capacity of insects to express systemic RNAi and eRNAi varies both within and between species (Baum & Roberts, 2014; Li et al., 2015a; Shukla et al., 2016; Sugahara et al., 2017).

Many factors affect the efficiency of gene silencing induced by eRNAi. Some are intrinsic properties of the insects themselves (genetic differences, feeding habits, etc.), but others correspond to the nature of dsRNA effector molecules and their state at the point of encounter/entry to the host. In this review, we first describe the mechanisms of RNAi in detail, highlight examples of its use in different pest species, and in the concluding section consider the factors affecting eRNAi, in an attempt to discover whether there are emergent properties that might be useful in the planning of SIT strategies.

## Mechanism of RNAi

### RNAi is facilitated through the canonical siRNA pathway, culminating in the degradation of target mRNA

**Figure 3:**
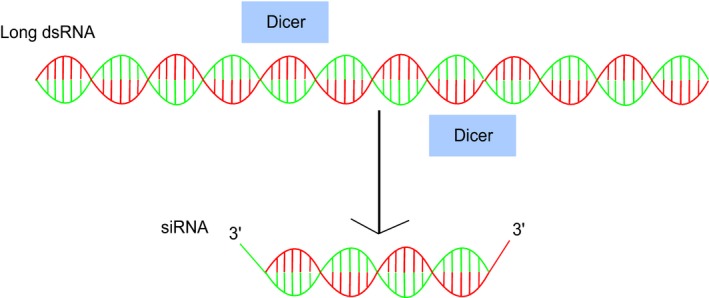
siRNA biogenesis. 21‐nt siRNA duplexes are cleaved from long, cytoplasmic dsRNA molecules by Dicer endonucleases. Two cuts are carried out by discrete Dicer RNAase III motifs, leaving short 3′ overhangs on each strand (Tomari & Zamore, 2005).

Small interfering RNAs (siRNAs) are short (ca. 21 nt) double‐stranded RNA molecules that are cleaved from long, cytoplasmic dsRNA transcripts (Figure 3). siRNAs belong to a large family of small non‐coding RNAs (ncRNAs) that facilitate different modes of gene silencing. ncRNA species include small interfering RNAs (siRNA), microRNAs (miRNA), PIWI‐interacting RNAs (piRNA), *trans*‐acting RNAs (tasiRNAs), repeat‐associated RNAs (rasiRNAs), and small‐scan RNAs (scnRNAs) (Kuramochi‐Miyagawa et al., 2001; Kim et al., 2009, 2015). While the origin and function of each ncRNA species is distinct (Bartel, 2004; Gasciolli et al., 2005; Babiarz et al., 2008; Kim et al., 2009) their actions are facilitated by homologous molecular mechanisms.

ncRNAs can only initiate gene silencing when bound to an Argonaute protein as part of an RNA‐induced silencing complex (RISC). When assembled in a RISC, exo‐siRNAs target viral mRNAs for knockdown as part of an immune response (Lan et al., 2016a,b). In contrast, endo‐siRNAs target endogenously transcribed mRNAs in order to achieve gene regulation (Babiarz et al., 2008; Okamura et al., 2008). exo‐siRNAs function through the canonical siRNA pathway, inducing cleavage of target mRNAs (Elbashir et al., 2001a; Song et al., 2004), while endo‐siRNAs inhibit the translation of target molecules (Hannon, 2002).

The canonical siRNA pathway requires the nucleotide sequences of siRNA molecules and their intended mRNA targets to exhibit almost perfect complementarity (Joseph & Osman, 2012). Imperfect homology may result in a mode of gene silencing other than mRNA cleavage (such as translational repression; Hu et al., 2010), which is associated with other ncRNA pathways. Perfect sequence homology is achievable in RNAi, as the target mRNA can usually be used to design effector dsRNA molecules with perfect matching. Therefore, the predominant mechanism of gene silencing induced by RNAi is mRNA degradation.

### siRNA biogenesis: Dicer

siRNAs are ubiquitous throughout the Eukaryota (Vaucheret, 2006; Fire, 2007), suggesting that defense to viral infection via the processing of long dsRNA is well conserved. Key effector molecules involved in siRNA biogenesis do vary in both form and function and have been demonstrated to be targets of viral suppression in honeybees (De Smet et al., 2017). Both endo‐ and exo‐siRNAs are cytoplasmically processed by Dicer, a member of the RNAase III endonuclease family (Hammond et al., 2000; Bernstein et al., 2001). RNAase III enzymes are defined as having two RNAase III endonuclease domains and a helicase domain (Sontheimer, 2005). As well as having three conserved RNAase III motifs, the Dicers also contain a conserved RNA‐binding PAZ domain (Yan et al., 2003) and a DUF283 domain with unknown function (Dlakić, 2006).

Dicer's PAZ domain binds the 3′ overhangs of long cytoplasmic dsRNA molecules. The captured dsRNA is then brought into contact with Dicer's two RNAase III domains, each of which cleaves (or dices) a particular strand of the molecule. Dicing produces 21‐nt siRNA duplexes with short (2 nt) overhangs at the 3′ end on each strand (Elbashir et al., 2001a).

Many organisms (including humans) express a single isoform of Dicer (Zhang et al., 2002). *Drosophila melanogaster* expresses two Dicer variants (Dicer 1 and Dicer 2) that are reported to function in discrete gene silencing pathways (Lee et al., 2004; Tomari et al., 2007), though many details are as yet unclear. Dicer 2 binds and degrades long dsRNA destined to become siRNA, whereas Dicer 1 binds pre‐miRNA hairpin loops of ca. 60 nt and cleaves them, creating functional miRNAs duplexes. Dicer 2 is also instrumental for processing of siRNA in the small brown planthopper, *Nilaparvata lugens* (Stål) (Lan et al., 2016a) and the zigzag leafhopper, *Recilia dorsalis* Motschulsky (Lan et al., 2016b).

### The RNA‐induced silencing complex (RISC)

RISCs are the functional components of all ncRNA‐mediated gene silencing pathways (Maniataki & Mourelatos, 2005; Rand et al., 2005; Hartig et al., 2007). RISCs can be defined as an Argonaute protein bound to a single strand of ncRNA. As there are several ncRNA and Argonaute species, the term RISC specifies a diverse group of ribonucleoprotein complexes.

Until complexed in a RISC, siRNAs have no effects upon gene expression. Formation of a RISC requires free siRNA to be captured by a RISC‐loading complex (RLC) and introduced to an Argonaute (Tomari et al., 2004; MacRae et al., 2008). After cleaving siRNA from long dsRNA, Dicer complexes with either transactivation response RNA‐binding protein (TRBP), or protein activator of PKR (PACT) to form the two human RLC variants (Haase et al., 2005; Lee et al., 2006, 2013; Lau et al., 2009). Variations in the 5′ terminal of TRBP and PACT may bias the binding affinities of human RLC variants toward either siRNA or miRNA, respectively (Lee et al., 2013). Dicer's participation in RISC loading is not required in all mammalian systems, as ΔDicer murine embryonic stem cells remain RLC competent (Murchison et al., 2005).

In *Drosophila* the canonical siRNA pathway utilizes an RLC formed by Dicer 2, R2D2 and TBP‐associated factor 11 (TAF11) (Liang et al., 2015). TAF11 is not necessary for RISC loading, as Dicer/R2D2 heterodimers form a competent RLC. Dicer and R2D2 bind opposite poles of siRNA before loading it into a RISC (Tomari et al., 2004). ∆R2D2 and ∆Dicer flies are therefore incapable of siRNA‐mediated gene silencing (Liu et al., 2006). Liang et al. (2015) suggest that an optimum RLC is formed when R2D2 and Dicer 2 form a tetrameric complex stabilized by TAF11. Tetrameric RLCs that include TAF11 display a 10‐fold increase in siRNA binding over Dicer/R2D2 heterodimers.

Dicer's role in siRNA biogenesis and RISC loading elicits potent gene silencing, in a manner which cannot yet be imitated precisely with artificially synthesized siRNA molecules. siRNAs that are enzymatically Diced from 240‐bp dsRNA constructs produce more effective gene silencing than artificially synthesized siRNA duplexes of equivalent sequence (Bolognesi et al., 2012). Whyard et al. (2009) also found that synthetic siRNA induced less potent RNAi than did enzymatically diced molecules.

### siRNAs associate with Argonaute proteins from the Ago Clade

Based on analysis of nucleotide sequence homology, the Argonaute proteins form two clades. The Ago clade is the largest and took its name from *Arabidopsis Ago1* mutants (Carmell et al., 2002). The Agos are bilobed proteins with a central PIWI endonuclease domain flanked by RNA‐binding PAZ (a feature shared with the Dicer enzymes), and MID domains at the N‐ and C‐terminals, respectively (Wang et al., 2008, 2009). A smaller Argonaute subclade (the PIWIs) was named for the *Drosophila* P‐element‐induced Wimpy testis protein (Aravin et al., 2006). Agos are expressed globally and associate with siRNA and miRNA to form RISCs. Until recently PIWIs (which associate with piRNAs) were thought to be restricted to the germline (Grishok et al., 2001; Morel et al., 2002; Tomari et al., 2007). However, evidence of their additional role in somatic gene silencing is now emerging (Morazzani et al., 2012; Schnettler et al., 2013).

Many organisms express a range of Ago proteins that associate with discrete ncRNA species. In *D*. *melanogaster*, for example, siRNA complexes with Ago2, miRNA associates with Ago1 (Tomari et al., 2007), and piRNA with PIWI proteins (Vagin et al., 2006; Malone et al., 2009). Although it is generally accepted that Ago2 is required for RNAi in the insects, silencing of Ago1 in *Leptinotarsa decemlineata* Say cells does inhibit gene silencing (Yoon et al., 2016). Humans, on the other hand, express four Ago proteins, all of which bind siRNA. However, only when complexed with Ago2 is siRNA capable of forming a functional RISC (Liu et al., 2004).

### Guide strand genesis

Ago2 degrades a single ‘passenger’ strand of each siRNA duplex presented by an RLC (Matranga et al., 2005; Rand et al., 2005; Leuschner et al., 2006; Wang et al., 2009). In humans and *D. melanogaster* Ago2 initiates the release of the passenger strand by cleavage, creating two single‐stranded molecules of 9 and 12 nt (Matranga et al., 2005; Noland & Doudna, 2013). In humans the fragmented passenger strand is then degraded in the cytoplasm by C3PO (Ye et al., 2011). It also appears that C3PO aids passenger strand digestion in *D. melanogaster* and may also enhance gene silencing through RISC activation (Liu et al., 2009). Once the passenger strand has been released, the remaining ‘guide’ strand complexes with Ago2 to form the RISC.

The thermodynamic properties of duplexed siRNA molecules appear to influence which strand is destined to be integrated into a RISC. The two strands of the siRNA duplex have to be separated from each other by helicases, which try to unwind the duplex from both ends. The ends can have different stability depending on the GC content on the last 3–5 base pairs and the strand that has the 5′ end at the less strongly paired end has a higher chance to become the guide strand (Khvorova et al., 2003; Schwarz et al., 2003; Tomari et al., 2004). Both human RLC variants are capable of sensing the thermodynamics of duplexed siRNA and reorientating the molecule prior to RISC loading (Noland et al., 2011; Noland & Doudna, 2013), which may facilitate strand selection by Argonaute. The Dicer/R2D2 RLC seen in *Drosophila* also configures siRNA according the thermodynamics of the molecule (Liu et al., 2006).

### RISCs utilize the guide strand to identify potential mRNA targets

Ago2's N‐terminal PAZ domain binds the 3′ end of the guide strand, whereas the C‐terminal MID domain binds the 5′ phosphate (Wang et al., 2008). The guide strand is orientated with its phosphate backbone toward Ago2's PIWI domain and the free nucleotides facing outwards. The RISC then utilizes the guide strand to scan cellular mRNA through Watson and Crick base pairing. Cognate mRNA, which base pairs with the guide, is targeted for knockdown (Filipowicz, 2005; Noland & Doudna, 2013). Each RISC is therefore capable of highly selective mRNA targeting based upon the nucleotide sequence of its intrinsic guide.

### Cleavage of targeted mRNA

In the final stage of RNAi, cleavage of targeted mRNA occurs in the region bound by the center of the guide strand between residues 10 and 11 (Elbashir et al., 2001b; Haley & Zamore, 2004). The resulting 5′ and 3′ mRNA fragments are then degraded by discrete cytoplasmic enzymes (Orban & Izaurralde, 2005). Within Ago2's PIWI domain is an aspartate‐aspartate‐glutamate (DDE) motif which is conserved in RNAase‐H related enzymes (Song et al., 2004). This motif is critical for mRNA degradation, as mutation of these residues results in loss of slicing ability (Liu et al., 2004).

## dsRNA as an experimental gene silencing device

In 1990, Napoli et al. (1990) developed petunias that expressed a hybrid chalcone synthase transgene (CHS). The authors predicted that expression of the transgene would supplement naturally occurring CHS and produce flowers with deep violet colouring (Napoli et al., 1990). Unexpectedly, 42% of the flowers exhibited an unpigmented, white phenotype. This led the authors to hypothesize that the transgene must somehow be inhibiting the expression of its naturally occurring orthologue.

Research into RNAi began following the work of Napoli et al. (1990). The first report of RNA being used to deliberately silence genes in an animal model came in 1995 when Guo & Kemphues (1995) injected *C. elegans* embryos with ssRNA designed to base pair with, and sequester, Par‐1 mRNA. Guo & Kemphues (1995) were successful in silencing Par‐1, but they were incorrect in their assumption that the underlying mechanism was triggered by ssRNA.

Using improved RNA preparation techniques, Fire et al. (1998) were able to show that Guo & Kemphues (1995) had contaminated their single‐stranded antisense RNAs with sense transcripts. Guo & Kemphues's (1995) ssRNAs had therefore base‐paired to form duplexes and entered the canonical siRNA pathway. Fire et al. (1998) were able to demonstrate that *C. elegans* when bathed in dsRNA silenced genes up to 100× more efficiently than when bathed in ssRNA. This experiment identified dsRNA as the critical effector molecules in previously described gene silencing experiments and was the first time dsRNA had purposefully been used to implement gene silencing. This finding was the starting point of all subsequent studies of RNAi.

## Implementation of eRNAi in pest species

As outlined briefly above, microinjection of dsRNA would not be a viable method for treating the large numbers of insects required for SIT. However, it is thought that exposure to eRNAi might provide a suitable alternative. The susceptibility of target species to eRNAi is critically important and has been reviewed in depth by Baum & Roberts (2014). However, insects that are naturally recalcitrant to eRNAi are not necessarily outside consideration for this type of gene silencing as, although yet to be demonstrated in an insect model, methods such as electroporation can also be used to deliver dsRNA – e.g., as described in tick eggs (Karim et al., 2010; Ruiz et al., 2015), nymphs, and larvae (Lu et al., 2015). Various options are outlined below.

### eRNAi delivery methods: larvae

To interrupt the sexual differentiation cascade in a manner that could be useful for SIT, RNAi must be implemented at the relevant critical developmental stages in eggs, embryos (Salvemini et al., 2009; Shukla & Palli, 2012; Liu et al., 2015), or early larvae (Whyard et al., 2015). Larvae are simple to target with eRNAi by ingestion as they eat steadily, volubly, and are generally less mobile than adults (hence can naturally take up dsRNA that is concentrated within local food sources). For aquatic larvae, dissolving dsRNA in solution and bathing the larvae within it, is the most common method of effecting gene silencing via eRNAi (Figueira‐Mansur et al., 2013; Singh et al., 2013; Whyard et al., 2015; Bona et al., 2016). dsRNA can be delivered to non‐aquatic larvae: (1) topically via droplet feeding (Toprak et al., 2013), (2) by inducing the larvae to feed upon dsRNA‐expressing transgenic plants (Xiong et al., 2013; Mamta et al., 2015; Tian et al., 2015; Hu et al., 2016), (3) by feeding larvae dsRNA‐expressing transgenic bacteria (Zhu et al., 2011; Yang & Han, 2014; Li et al., 2015c), and (4) by feeding larvae naked dsRNA overlaid onto an artificial diet (Asokan et al., 2014; Yang & Han, 2014; Hu et al., 2016). Non‐aquatic larvae, or those that develop in relatively anoxic conditions, can also be bathed in dsRNA solution, but the timing of exposure is critical to avoid drowning (Whyard et al., 2009). Choi et al. (2012) also report delivery of dsRNA via parental feeding in a study in which nurse ant workers were fed with dsRNA that was then passed to larvae via regurgitation.

### eRNAi delivery methods: adults

Genes that can induce sterility when knocked down can be targeted in adult insects for use with SIT. eRNAi has been demonstrated to successfully achieve gene silencing in adults following ingestion of: (1) dsRNA‐expressing transgenic plants (Coleman et al., 2015; Tzin et al., 2015; Malik et al., 2016), (2) dsRNA‐expressing transgenic bacteria (Li et al., 2011; Taracena et al., 2015; Whitten et al., 2015), (3) dsRNA dissolved in solution (Coy et al., 2012; Ratzka et al., 2013; Shim et al., 2015), and (4) naked dsRNA overlaid on diet (Yi et al., 2014; Zheng et al., 2015). In addition, topical application to adults of dsRNA (Pridgeon et al., 2008; Killiny et al., 2014; Amiri et al., 2015) and infection with transgenic fungi (Chen et al., 2015) are reported. All methods have the potential for use in SIT development. However, the use of transgenic plants may be limited by the feeding habits of target pests, and fungi also need to be tested for their potential to infect unintended secondary targets.

An important consideration for eRNAi silencing for insect control is the feasibility of producing and delivering the required amount of dsRNA. Both in vitro and in vivo methods for producing dsRNA for insect control have been tested, as described below.

### In vitro dsRNA synthesis

The T7 RNA polymerase (from the T7 bacteriophage) is a highly selective enzyme that enables rapid synthesis of RNA sequences (Tabor, 2001). For in vitro production of dsRNA, linear DNA sequences that code for both sense and antisense RNA transcripts flanked by the 20‐nt T7 promoter are transcribed by incubation with T7 polymerase (Singh et al., 2013; Liu et al., 2015; Shim et al., 2015; Whyard et al., 2015). Cognate ssRNA transcripts then base pair to form dsRNA that can be used for eRNAi experiments.

### In vivo dsRNA synthesis by bacteria

dsRNA can be synthesized in vivo by bacteria themselves using transgenic HT115 *Escherichia coli* (Migula) Castellani & Chalmers (Kamath et al., 2001). The HT115 genome has been modified to be RNase deficient and to contain a T7 polymerase under the control of lactose regulatory elements. Generally, target sequences flanked by two T7 promoters at each side are introduced to L4440 plasmid vectors by ligation. The plasmid is then transformed into HT115 bacteria and target DNA sequences are transcribed by T7 polymerases induced by the allolactose mimic IPTG (Whyard et al., 2009, 2015; Zhu et al., 2011; Yang & Han, 2014; Taracena et al., 2015). A limitation of this method is that, once introduced to target insects, the effect is transient as the HT115 bacteria fail to colonize the gut and become established in the insect gut microbiome.

Modified symbiotic bacteria have recently been utilized as an alternative to HT115 *E. coli* (Whitten et al., 2015). In this study, microbes from the microbiome of target species were reprogrammed to have similar properties to HT115 (in that they were RNAse‐deficient), but RNA sequences were constitutively active rather than being inducible. These symbiotic bacteria were able to repopulate the gut of target insects and induce a long term silencing effect. These results suggest that there is great potential to genetically engineer naturally occurring bacteria in the gut microbiomes of pest species for control purposes.

### In vivo dsRNA synthesis by plants

The nuclear genome of plants can be modified using *Agrobacterium tumefaciens* Smith & Townsend (De Block et al., 1984; Horsch et al., 1984) to express non‐endogenous dsRNA. dsRNA constructs can be expressed as either a single sequence which forms a long hairpin (hpRNA) (Xiong et al., 2013; Guo et al., 2014; Mamta et al., 2015), or two separate complementary transcripts which base pair in the cytoplasm (Kumar et al., 2012). However, transfer of target sequences into the genome of plant hosts is unpredictable and dsRNA abundance in similarly prepared transgenic plants can vary by up to 900% (Tian et al., 2015).

The genome of plant chloroplasts can also be programmed to synthesize non‐endogenous dsRNA (Jin et al., 2015; Zhang et al., 2015a). Due to the prodigious metabolic output of these organelles they are capable of rapid production of large amounts of effector dsRNA molecules. As for the gut microbiota, there is potential for engineering chloroplasts in this manner for application to insects through eRNAi. In the next sections we consider the design features of dsRNAs that may render them useful for control.

### Optimum dsRNA construct design: length and GC content

There is a minimum length threshold (MLT) at which dsRNA can induce eRNAi. The MLT has been demonstrated to be ca. 60 bp in several insects (Bolognesi et al., 2012; Miller et al., 2012; Ivashuta et al., 2015), although Miyata et al. (2014) reported an MLT of ca. 100 bp. The MLT for eRNAi is defined by the minimum length of dsRNA that can be absorbed by the intestine. However, distal tissues may be capable of absorbing shorter transcripts. Ivashuta et al. (2015) report an MLT of 60 bp in *Diabrotica virgifera virgifera* LeConte, and the uptake of a 21‐bp siRNAs by the fat body of this insect.

Once the MLT has been met, dsRNA construct length is not an accurate predictor of RNAi potency, as constructs of similar length can elicit diverse silencing effects (Toprak et al., 2013; Asokan et al., 2014). Most RNAi research in insects is carried out using dsRNA constructs of between 200–500 bp (Table 1), although successful silencing has been achieved using constructs of up to 1 800 bp (Baum et al., 2007).

**Table 1:** A selection of examples of the effectiveness of eRNAi across diverse insect taxa. The studies included highlight variation in the strength of silencing that can be induced via different methods of dsRNA delivery in various taxa and the differences observed in temporal effects

	Species	dsRNA delivery method	Construct length/bp	Genes targeted	Temporal effects of RNAi	Quantification of gene knockdown	Reference
Hymenoptera	*Camponotus floridanus* (Buckley)	Adults fed dsRNA dissolved in sucrose solution for up to 15 days	≈ 400	Adults: *peptidoglycan recognition protein (PGRP‐LB)*	Maximum gene silencing after 5 days	≈ 100%	Ratzka et al. (2013)
	*Solenopsis invicta* Buren	dsRNA fed to larvae via nurse workers for 12 days	496	*Pheromone biosynthesis activating neuropeptide (PBAN)*	After 21 days mortality increased by 50%	Not known	Choi et al. (2012)
Coleoptera	*Diabrotica virgifera virgifera*	Embryos targeted via parental RNAi; adults fed dsRNA overlaid on artificial diet for 10 days	352, 405	*Brahma (bhm)* and *Hunchback (hb)*	10 days after egg laying hatching rates were 0% (*bhm*) and 2.4% (*hb*)	99% silencing of brahma in eggs; 85.3% silencing of hunchback in eggs	Khajuria et al. (2015)
	*Leptinotarsa decemlineata*	L2 larvae fed dsRNA or transgenic bacteria overlaid on potato leaves	200–400	5× *Housekeeping* genes	Bacteria‐treated larvae exhibit higher mortality than naked dsRNA treatment, 12 days after feeding	Bacteria‐treated: 59–91%; dsRNA: 61–93% (both gene‐dependent)	Zhu et al. (2011)
	*D. v. virgifera*	L1 larvae fed dsRNA overlaid on diet for up to 8 days	250, 500, 750, 1000	*Ebony* and *laccase 2 (lac2)*	≈ 90% silencing after 2 days	Lac2 ≈ 100%; ebony ≈ 95%	Miyata et al. (2014)
Hemiptera	*Myzus persicae* (Sulzer)	Adults fed on transgenic plants for up to16 days	Not known	*Receptor of Activated Kinase C (Rack1), MpPIntO2* & *MpC002*	Maximum effect seen after 8 days. Treated insects demonstrate gene silencing for up to 6 days post‐feeding. Progeny of treated insects demonstrate gene silencing for up to10 days post‐feeding	≈ 70% for all genes	Coleman et al. (2015)
	*M. persicae/Bactericera cockerelli (Šulc)*	Adults fed on transgenic plants for up to 8 days	250–500	*Aquaporin (AQP), sucrase (SUC),* and *sugar transporter (ST4)*	*B. cockerelli*: osmotic pressure of haemolymph varied significantly for 8 days; *M. persicae*: gene expression altered for 7 days	*B. cockerelli*: no significant silencing of any genes; *M. persicae*: significant silencing of all genes	Tzin et al. (2015)
	*Rhodnius prolixus* Stål	Adult females fed ≈ 154.2 ng of dsRNA incorporated in transgenic bacteria	375, 453	*Rhodnius heme‐binding protein (RHBP)* and *catalase (CAT)*	*RHBP*: peak silencing after 3 days of feeding, no effect 10 days after feeding; *CAT*: peak silencing after 5 days of feeding	RHBP: 99.6%; CAT: 96% silencing	Taracena et al. (2015)
	*Diaphorina citri* Kuwayama	Topical application of dsRNA dissolved in solution to adults	Not known	5× *Cytochrome p450* genes	Significant lack of protein 8 days after treatment	50–100% silencing (gene dependent)	Killiny et al. (2014)
	*Bemisia tabaci* (Gennadius)	Adults fed dsRNA dissolved in sucrose solution for 1 day.	279	*Heat shock protein 70 (hsp70)*	Gene silencing for up to 3 days after feeding.	100% silencing	Shim et al. (2015)
Lepidoptera	*Mamestra configurata* Walker	dsRNA applied topically to L1 or L4 larvae, which are then fed with leaf discs overlaid with dsRNA for up to 2 days	500	*Chitin deacetylase 1 (CDA1)*	Larvae silence *CDA1 1* (L1) and 1.5 (L4) days after treatment	L1: ≈100% silencing; L4: silencing not quantified	Toprak et al. (2013)
	*Helicoverpa armigera*	Larvae fed transgenic plants or bacteria for 7 days	400–600	Larvae: *molt regulating transcription factor (HR3)*	2‐day latency of effect; larvae fed on transgenic plants have 70% less mass than controls after 6 days	Bacteria: ≈ 80%; plants: ≈ 85%	Xiong et al. (2013)
	*H. armigera*	Larvae fed (all instars) transgenic bacteria and dsRNA overlaid on artificial diet	562, 450	*Ultraspiracle protein (Ups)* and *ecdysone receptor gene (EcR)*	Surviving larvae were assayed by qRT‐PCR 5 days after treatment	60% silencing of USP via continuous bacterial feeding	Yang & Han (2014)
Diptera	*Aedes aegypti*	Topical application of dsRNA to adult females	252, 436, 556	3× *Inhibitor of apoptosis (IAP)* genes	Mosquitos assessed 1 day after treatment	33–87.5% silencing (gene dependent)	Pridgeon et al. (2008)
	*Anopheles gambiae* Giles	L3 larvae fed dsRNA complexed in chitosan nanoparticles	Not known	*Chitin synthase 1 (CHS1)* and *chitin synthase 2 (CHS2)*	After 4 days of treatment with *CHS1* (but not *CHS2*), larvae silenced both intended target genes	CHS1: 62.8% silencing of CHS1 and 57.9% of CHS2; CHS2: 63.4%	Zhang et al. (2010)
	*Bactrocera dorsalis*	Adults fed dsRNA overlaid on artificial diet until death	Not known	*Sex peptide receptor (spr)*	Mean life span reduced by 26 days in treated flies	52%	Zheng et al. (2015)
	*B. dorsalis*	Adults fed dsRNA overlaid on artificial diet for up to 14 days	297, 394	*Odorant receptor (Bdor)* and *odorant coreceptor (Orco)*; targets silenced individually and simultaneously	4‐day latency of effect, peak effect after 7 days	70% when both targets applied simultaneously	Yi et al. (2014)
	*Drosophila melanogaster*	L1 larvae soaked in PBS buffer containing dsRNA bound in liposomal vectors for 1 h	≈ 400	*β‐Glucuronidase gene (gus)*	Flies assessed 1 day after feeding	Lipofamectine 2000 vectors: 53%	Whyard et al. (2009)

The GC content of dsRNA negatively correlates with eRNAi efficiency (Reynolds et al., 2004; Chan et al., 2009). GC bonds are more stable than AU bonds and less prone to unwinding by Dicer's helicase domain.

### Optimum dsRNA construct design: target sequence

Specific nucleotide sequences within targeted mRNAs are intrinsically susceptible to RISC cleavage. Bolognesi et al. (2012) report that a single homologous 21‐bp sequence can silence mRNA as efficiently as a 60‐bp construct of 100% homology. RNAi potency is therefore governed partly by the quality of 21‐nt sequences contained within dsRNA constructs.

Discrete dsRNA constructs targeting different regions of the same mRNA molecule have been demonstrated to elicit variable silencing effects (Xiong et al., 2013; Tian et al., 2015). Neither the pole nor the mid‐region of mRNA appears to be intrinsically prone to cleavage, as susceptibility has been reported at various regions of targeted molecules (Mao & Zeng, 2012; Xiong et al., 2013). Asokan et al. (2014) targeted five unrelated mRNAs in *Helicoverpa armigera* (Hübner) and observed variation in silencing efficiency from 21 to 95%.

Sfold software (Ding et al., 2005) is reported to predict the regional susceptibility of mRNA to RISC cleavage prior to construct design, though data from Xiong et al. (2013) showed predictive success to be variable. Regional susceptibility to cleavage is likely to be defined largely by the secondary structure of the target molecule. RNAi potency is inversely proportional to the amount of hydrogen bonds formed between target and non‐target sequences (Luo & Chang, 2004). This implies that targets that are tightly bonded to local sequences are unavailable to the RISC complex, rendering gene silencing inefficient.

### Optimum dsRNA construct design: off‐target effects

Non‐target mRNA with precise homology to the guide strand of a RISC will inevitably be targeted and degraded. Hence, it is important to consider the potential for inadvertently silencing any other genes within the same or different species that contain these sequences. Such off‐target effects (OTEs) can affect endogenous genes of the experimental target (Kulkarni et al., 2006; Ma et al., 2006; Zhang et al., 2010; Toprak et al., 2013), predators of the target species following consumption (Garbian et al., 2012), and closely related species (Zhu et al., 2012). Singh et al. (2013) found that sequences of continuous homology greater than 19 nt were required to induce OTEs in target (*Aedes*) and non‐target (*Drosophila*) species. Ulrich et al. (2015) report that OTEs are equally probable in conserved and non‐conserved amino acid sequences.

It is important to minimize the potential for OTEs by careful design of dsRNAs. ‘E‐RNAi’ (Horn & Boutros, 2010) and ‘SnapDragon’ (Harvard Medical School, 2016) are examples of software that automatically design dsRNAs for use with RNAi and search for OTEs in a selection of well‐referenced genomes. If dsRNAs are designed manually then dsCheck (Naito et al., 2005) can be used to predict potential OTEs. However, it is difficult to fully anticipate off‐target matching for sequences that have not yet been described.

### Genetic attributes which facilitate eRNAi – the *Caenorhabditis elegans* example

The nematode *Caenorhabditis elegans* (Maupas) was the first species in which RNAi was successfully implemented (Fire et al., 1998) and has been used extensively to investigate genetic mechanisms underlying gene silencing. This research has uncovered five systemic interference defective (SID) genes that facilitate RNAi (Feinberg & Hunter, 2003; Jose & Hunter, 2007; Jose et al., 2012). SID2 is an intestinal transmembrane protein that is thought to independently endocytose vesicular dsRNA from the gut lumen, before it is processed in the cytoplasm by Dicer (McEwan et al., 2012). SID2 proteins are essential for eRNAi in *C. elegans* as they allow for passage of ingested dsRNA molecules and enable eRNAi when transgenically expressed in recalcitrant species (Winston et al., 2007). SID1 is a ubiquitously expressed transmembrane protein that is essential for systemic RNAi (Winston et al., 2002; Jose et al., 2009). SID1's precise mode of action is yet to be elucidated, but it transmits silencing signals between cells, either in the form of long dsRNAs, free siRNAs, or siRNA bound by RISCs.

In *C. elegans*, RNAi is propagated by RNA‐dependent RNA polymerases (RdRp) (Sijen et al., 2001). RdRps bind primary cytoplasmic RNA transcripts and utilize them to synthesize secondary dsRNAs, which then re‐enter the siRNA pathway extending the period of gene knockdown (Pak & Fire, 2007). Many viruses also encode RdRps, which allow for the proliferation of viral RNAs (Pan et al., 2016).

The wealth of information about the mechanism of RNAi gained from the study of *C. elegans* has been of great use for elucidating homologous mechanisms in pest species. The transfer of enabling mechanisms, such as SID1 gene functionality, to species that lack them has also provided insights into gene silencing mechanisms that might exist in some pest species.

### Genetic attributes that facilitate eRNAi in the insects

Orthologues of SID genes are found across insect taxa but are absent from the Diptera (Huvenne & Smagghe, 2010), members of which represent some of the world's most significant agricultural and medical pests. An orthologue of SID1 facilitates systemic RNAi in *Apis mellifera* L. (Aronstein et al., 2006) and Miyata et al. (2014) suggest that two SID orthologues are involved in, if not essential to, eRNAi in *D. v. virgifera*. On this basis, the presence or absence of SIDs has been used by some researchers to predict the capacity of different insect species to effect gene silencing via eRNAi (Tomoyasu et al., 2008; Tian et al., 2009). However, the presence of SIDs is not fully predictive of eRNAi potential – several studies have demonstrated that dipterans, which lack SIDs as noted above, are eRNAi competent (Table 1). In contrast, *Bombyx mori* (L.) possesses three SID orthologues, yet is not competent for eRNAi (Li et al., 2015d).

SIDs are not the only molecules that contribute to dsRNA uptake as eRNAi is facilitated by endocytic pathways in some insect species. For example, *Bactrocera dorsalis* (Hendel) (Li et al., 2015b) and *Tribolium castaneum* (Herbst) (Xiao et al., 2015) are refractory to eRNAi following challenge with the endocytic inhibitor Bafilomycin A1 (Xu et al., 2003). Refractoriness to eRNAi can also be induced in *Bactrocera* and *Tribolium* if orthologues of the *chc* (*clathrin heavy chain*) gene (Bazinet et al., 1993) are downregulated (Li et al., 2015b; Xiao et al., 2015). It has recently been suggested that SIDs and endocytic mediators synergistically facilitate eRNAi in *L. decemlineata* (Cappelle et al., 2016). Scavenger receptors have also been demonstrated to facilitate systemic RNAi in *D. melanogaster* (Saleh et al., 2009) and eRNAi in *Schistocerca gregaria* Forsskål (Wynant et al., 2014).

Recent evidence suggests that an intracellular mode of dsRNA degradation, other than the siRNA pathway, may exist in some insect species (Shukla et al., 2016). Shukla et al. (2016) demonstrated that cell lines of *L. decemlineata* and *Spodoptera frugiperda* (JE Smith) were both capable of dsRNA uptake, although only the cells of *Leptinotarsa* produced 21‐bp siRNA‐like transcripts. Apparently dsRNA was degraded in endosomes within the cells of *Spodoptera*, as demonstrated by pH‐induced fluorescence of CypHer5E‐labelled molecules. Accordingly, Yoon et al. (2016) report that dsRNA may escape endosomes through acidification in *L. decemlineata*, facilitating induction of the RNAi process in that insect. These types of practical studies, along with comparative transcriptomic analyses (such as Swevers et al., 2013), may help us to understand the divergence in eRNAi potency between insects.

Orthologues of RdRps have not been reported in the Insecta. Several insects can nevertheless exhibit sustained RNAi for prolonged periods (Paim et al., 2013; Coleman et al., 2015; Khajuria et al., 2015), suggesting a system of signal amplification. Hemipterans appear to have a robust RNAi amplification system, as gene silencing has been demonstrated for 4 days (Rebijith et al., 2016), 6 days (Coleman et al., 2015), and 8 days (Tzin et al., 2015) after feeding with dsRNA. Coleman et al. (2015) also report that nymphs born from RNA‐treated mothers exhibited RNAi for 10 days post‐feeding, suggesting that genetic variation between life‐history stages might influence signal amplification.

Insects that produce RNAses in salivary and midgut secretions or in haemolymph can degrade dsRNAs and thus limit the extent of gene silencing (Allen & Walker, 2012; Liu et al., 2012; Garbutt et al., 2013; Yang & Han, 2014; Shukla et al., 2016). Yang & Han (2014) suggest that RNAi is more efficient in *H. armigera* if dsRNAs are encapsulated in bacteria when they traverse the gut, as this bypasses potent digestive RNAases in midgut secretions. Das et al. (2015) present a similar idea, but suggest that vectoring dsRNA in carbon quantum dot nanoparticles provides protection, not from nucleolytic enzymes, but from damage incurred through extreme pH in the alimentary canal of *A. aegypti*.

## Conclusion

In this review we have described the detailed mechanisms underlying gene silencing by dsRNA, and considered the use of this approach for use with SIT. Currently, the available data are scant and insufficient to design all aspects of eRNAi studies in pest species in a predictive context. We have emphasized several factors that must be considered in the design and implementation of such techniques (Table 2) in order to try to address these omissions.

**Table 2:** Practical considerations when designing eRNAi‐based SIT strategies

eRNAi is dose‐dependent.	The potency of gene silencing correlates with dsRNA concentration and period of exposure (Zhou et al., 2008; Tian et al., 2009, 2015; Singh et al., 2013; Asokan et al., 2014; Yu et al., 2014; Li et al., 2015b; Ulrich et al., 2015; Whyard et al., 2015; Rebijith et al., 2016). A period of latency between dsRNA administration and gene silencing is common in larvae and adults, which is consistent with a threshold effect. Reported latency periods include: 12 h in *Helicoverpa armigera* (Tian et al., 2015) and *Aedes aegypti* (Coy et al., 2012), 24 h in *Mamestra configurata* (Toprak et al., 2013) and *Bactrocera dorsalis* (Zheng et al., 2015), 7 days in *Spodoptera exigua* (Tian et al., 2009), and 12 days in *Solenopsis invicta* (Choi et al., 2012). It is of note that Choi et al. (2012) did not feed dsRNA to insects directly but via a secondary worker individual, and this may have diluted dsRNA somewhat leading to an extended latency period.
Taxonomy cannot be used to reliably predict sensitivity to eRNAi or the latency period between dsRNA uptake and gene silencing.	Equivalent eRNAi methods can produce disparate results even when different biotypes (Li et al., 2015a), or subpopulations (Sugahara et al., 2017) of the same species are targeted. Within the Lepidoptera, *H. armigera* is capable of eliciting a robust eRNAi response (Xiong et al., 2013), but *Spodoptera frugiperda* is recalcitrant to eRNAi (Ivashuta et al., 2015). Working on hemipteran and dipteran models, Coleman et al. (2015) and Yi et al. (2014) observed a 4‐day period of latency between dsRNA administration and gene silencing. Another dipteran (*A. aegypti*) exhibited gene silencing after 12 h of feeding with dsRNA in solution (Coy et al., 2012). These discrepancies may be eliminated if equivalent feeding protocols are used.
The sensitivity of a gene to RNAi has not been fully assessed unless the entire mRNA molecule has been targeted for knockdown.	When all variables remain constant, variation in RNAi potency is likely to be due to regional susceptibility of mRNAs to cleavage. The strength of gene knockdown can vary greatly when multiple genes are targeted in the same insect using identical methods (Pridgeon et al., 2008; Li et al., 2011; Singh et al., 2013; Toprak et al., 2013; Killiny et al., 2014; Taracena et al., 2015).
Insects may become more tolerant to dsRNA with aging.	A diminution in the efficiency of RNAi with age has been suggested by Tian et al. (2015) and is supported by evidence that silencing appears more efficient in neonates than in late stage larvae (Zhu et al., 2011; Toprak et al., 2013). Furthermore, Coleman et al.'s (2015) observation that silencing is longer lived in nymphs than in adults suggests that fully developed insects are less sensitive to dsRNA.
Optimum eRNAi delivery methods must be determined by trial and error in most insect species.	Yang & Han (2014) found that feeding *H. armigera* with transgenic bacteria induced more efficient eRNAi than feeding with naked dsRNA. However, naked dsRNA elicited more robust gene silencing than did bacterial feeding in *B. dorsalis* (Li et al., 2011). Zhu et al. (2011) report that three of five genes were knocked down more efficiently using a bacterial system in *Leptinotarsa decemlineata*, but that the remaining two were more efficiently silenced by naked dsRNA.
When inducing eRNAi the capacity for systemic RNAi is critical if target genes lie beyond gut tissue.	The systemic RNAi capacity of various insects has been assessed by targeting *chitin synthase* genes specific to the exoskeleton (Tian et al., 2009; Zhang et al., 2010; Singh et al., 2013). Other examples of studies that targeted genes distal to gut tissue include the silencing of *ebony* in *Diabrotica virgifera vigifera* (Miyata et al., 2014) and *Rhodnius* heme binding protein (RHBP) in *Rhodnius prolixus* (Taracena et al., 2015). Ivashuta et al. (2015) suggest that systemic RNAi in *D. v. virgifera* is facilitated by transport of dsRNAs of >60 bp long.
Parental RNAi requires further analysis to determine whether it can be effective for SIT.	A robust systemic RNAi response enables the silencing of genes in germ cells (parental RNAi; pRNAi). When germ cells are affected by pRNAi, gene expression can be limited in zygotes and developing insects (Zwier et al., 2012; Paim et al., 2013; Coleman et al., 2015; Khajuria et al., 2015). A simple application of pRNAi in pest management would be to reduce future insect populations via embryonic lethal gene silencing (Khajuria et al., 2015). For use with SIT, sexual differentiation genes could be targeted in the mothers of target insects (Shukla & Palli, 2012). dsRNA delivery methods may drastically affect the potency of pRNAi. Zheng et al. (2015) report that eRNAi silencing of *sex peptide receptor* in *B. dorsalis* limited eclosion rates of their progeny, whereas Peng et al. (2015) describe that silencing the *transformer* gene by microinjection in this species has no effect on progeny. The disparity in pRNAi efficiency between these delivery methods might be due to the fact that eRNAi would have consistently supplied flies with dsRNA during the development of germ cells, whereas expression of *transformer* would have been only transiently reduced by microinjection.
Insects may become less sensitive to dsRNA over time.	Working with *B. dorsalis*, Li et al. (2015b) demonstrated that eRNAi potency was reduced following a series of exposures to dsRNA. The effect was dose‐dependent, was not gene specific, and lasted for up to 20 days following primary exposure. Refractoriness only occurred following targeting of endogenous genes, which suggests a role in immune priming. Li et al. (2015b) suggest that flies may become refractory to dsRNA when genes that mediate endocytosis are downregulated. *Bactrocera dorsalis* has also been reported to upregulate the expression of target genes following exposure to dsRNA (Li et al., 2011). Therefore, reduced eRNAi potency may be due to synergistic overexpression of target genes along with downregulation of endocytic mediators.
Endocytic pathways and SID transport proteins may work synergistically in eRNAi.	Both endocytosis and SID mediated dsRNA transport facilitate eRNAi in *L. decemlineata* (Cappelle et al., 2016).
Insects may become more sensitive to eRNAi if dsRNA is vectored in nanoparticles. The performance of various nanoparticle technologies for use with RNAi is reviewed in Liao et al. (2016).	Nanoparticle‐based delivery of dsRNA may serve dual purposes: (1) enhancing passage of dsRNA across the gut, and (2) prolonging the effect of RNAi via slow release of dsRNA. Whyard et al. (2009) utilized Lipofectamine 2000 and Cellfectin liposomal nanoparticles to successfully vector dsRNA to *Drosophila melanogaster*, even though this species is reported as eRNAi incompetent. Liposomal vectoring achieved ca. 50% gene knockdown. Recently, *Drosophila suzukii* (Matsumura) has also been reported as recalcitrant to feeding with naked dsRNA, but sensitive if molecules are vectored in liposomes (Taning et al., 2016). Chitosan nanoparticles can vector molecules to *Anopheles gambiae* and *A. aegypti* (Zhang et al., 2010, 2015b; Mysore et al., 2015), although mosquito larvae (Figueira‐Mansur et al., 2013; Singh et al., 2013; Whyard et al., 2015) and adults (Pridgeon et al., 2008; Coy et al., 2012) also demonstrate eRNAi when exposed to naked dsRNA. Potent eRNAi has been demonstrated in *A. aegypti* larvae using carbon quantum dot (CQD) nanoparticles (Das et al., 2015).
Data regarding the simultaneous knockdown of genes via administration of multiple dsRNAs are conflicting.	Simultaneous silencing of genes has been reported to enhance the potency of RNAi in *A. aegypti* using a bacterial feeding approach (Whyard et al., 2015). A plant feeding study of *Myzus persicae* and *Bactericera cockerelli* also suggested that targeting genes simultaneously may induce a synergistic effect (Tzin et al., 2015). Zhang et al. (2015a) and Ulrich et al. (2015) report simultaneous silencing in coleopteran models actually dilutes the potency of RNAi. When feeding combinations of dsRNAs to *D. suzukii*, Taning et al. (2016) found the potency of RNAi was enhanced for some target gene combinations but not others.

Knowledge of eRNAi in key areas, such as the most basic mechanisms that enable insects to acquire dsRNA from their environment, is lacking. SIDs and endocytosis both play roles individually and synergistically, but overall the picture of their modes of action is far from clear. Packaging of dsRNA in intermediate carriers such as bacteria or nanoparticles may overcome refractoriness to eRNAi in some cases. However, certain insects may remain refractory to eRNAi even if dsRNA is successfully packaged and transported across the gut, as discrete modes of dsRNA degradation such as endosomal acquisition may mitigate the silencing process.

SIT strategies rely on mass rearing, which in some cases has knock‐on effects for insect quality and performance (Sørensen et al., 2012). The consequences of mass rearing manifest differently across taxa, which is why Chambers (1977) suggested comprehensive quality control measures for all such programs. SIT individuals generated (by any means) from mass‐reared populations are likely to perform differently than untreated controls. All SIT techniques should be judged according to the performance of individuals in the field, which very often will differ to those in the laboratory or factory (Mayer et al., 1998; Carvalho et al., 2015). The goal of eRNAi‐SIT is to produce mass‐reared insects that are at least equal in quality to the currently available alternatives.

The potential of an eRNAi approach is being increasingly realized and groups such as the SITplus partnership are developing this technology to combat the spread of pest insects (CSIRO, 2015). The production of dsRNA for large‐scale eRNAi treatments may be expensive. HT115 *E. coli* (see ‘In vivo dsRNA synthesis’ section above) may represent the most viable option currently available, but economy of scale does present a challenge that needs to be addressed.

As suggested by the transformer‐2/innexin‐5 model, eRNAi could be used in the future to successfully implement gene silencing, and create insects for application of SIT in the field. There is evidence that other gene targets could also be utilized in an eRNAi‐SIT system. A possible outcome when the sexual differentiation cascade is targeted with RNAi is a combination of arrested phenotypic female development in some individuals, and sex‐reversal in others. This has been demonstrated by Shukla & Palli (2012) in *T. castaneum*, where parental RNAi of transformer produced a cohort of 91.1% males, 8.9% pseudomales, and 0% females. This outcome is not optimal for SIT as nearly half the population was lost, but it did produce a compatible male‐only cohort. More recently, eRNAi of spermatogenic targets has been demonstrated to induce sterility by up to 60% in *B. dorsalis* while maintaining mating competitiveness (Dong et al., 2016).
